# Influence of impurities on the use of Fe-based powder as sustainable fuel

**DOI:** 10.1098/rsta.2023.0236

**Published:** 2024-11-04

**Authors:** Laurine Choisez, Marie-Aline Van Ende, Zakarie Bruyr, Francesco Contino, Pascal J. Jacques

**Affiliations:** ^1^ UCLouvain, Institute of Mechanics, Materials and Civil Engineering, IMAP, Place Sainte Barbe, 2, Louvain-la-Neuve B-1348, Belgium; ^2^ Department of Materials Science and Engineering, Seoul National University and Research Institute for Advanced Materials, 1 Gwanak-ro, Gwanak-gu, Seoul 08826, Republic of Korea; ^3^ Department of Mechanical Engineering, Eindhoven University of Technology, P.O. Box 513, Eindhoven, MB NL-5600, The Netherlands

**Keywords:** metal fuel, sustainable energy carrier, iron combustion, alloy design, thermodynamic simulation, adiabatic combustion

## Abstract

Sustainable energy production, inherently transient and non-uniformly distributed around the world, requires the rapid development of sustainable energy storage technologies. Recently, pure iron powder was proposed as a high-energy density carrier. While promising, challenges are faced, such as nanoparticle emissions, micro-explosions or cavitation. In this work, a screening of the impact of the most common impurities in iron sources on these mechanisms was conducted through purely thermodynamic simulations. Two idealized models were considered to obtain a range of plausible flame temperatures and emitted gases when considering a purely diffusive regime in standard conditions and stoichiometric air–fuel mixture. The flame temperature and iron evaporation are increasing with the specific energy. A strong evaporation of C, S, Mo, Cu and P is also expected. Most impurities are predicted to decrease cavitation, except for Mn and MnO. The regeneration process by hydrogen-based direct reduction in fluidized bed reactors is also discussed. MgO and CaO are the most promising additions in terms of reducing nanoparticles and porosities, as well as to improve the fluidization and reduction kinetics of the combusted products. The potential of Fe powder as sustainable fuel, already very promising, could be further improved by the addition of selectively chosen impurities.

This article is part of the discussion meeting issue 'Sustainable metals: science and systems'.

## 1. Introduction

Defossilization of energy carriers is urgently required to tackle global warming, as it accounts for more than 70% of global greenhouse gas emissions [1]. Sustainable storage technologies are key to overcoming the spatiotemporal intermittence of renewable energy production based on solar and wind energies. Recently, metal fuels have been introduced as a new carbon-free, energy-dense and sustainable energy carrier [2]. In this concept, heat is generated by the combustion of the metallic powder, i.e. its high-temperature exothermic oxidation, producing solid oxide particles as a product of the reaction. These oxidized particles can then subsequently be regenerated by reducing the combustion products back to their metallic form. During this reduction process, the excess of renewable energy is therefore stored, and is then released during the high-temperature oxidation of the metal particles. Among the different metals considered, iron is particularly suited for this application because of its large abundance and availability. Moreover, iron is the only metal known with a flame temperature lower than its evaporation temperature (3140K) and the evaporation temperature of its oxides (3396K) [3] in standard conditions (atmospheric pressure) in air (21% oxygen concentration), limiting nanoparticle emissions compared with other considered metal fuels [3]. Because most of iron combustion occurs in the liquid state in standard conditions and not in the gaseous state, the combustion products keep a similar size as the initial iron particles, facilitating a direct cycling between combustion and reduction of the combusted products.

Techno-economic assessments of the use of iron powder as high energy density fuel have been conducted by several research groups, estimating a power-to-power overall efficiency of 26–33% [4] and 19–29% [5] (including the regeneration process by green hydrogen electrolysis and hydrogen-based direct reduction, transport of the powders between North Africa and Northern Europe and steam power plants). This efficiency is similar to the one of hydrogen when transported over similar distances and burned for energy release (23–38% from Debiagi *et al*. [4] and 16–32% from Neumann *et al*. [5]). Yet iron presents the main advantage of a larger energy density for efficient storage, higher safety and stability over time.

Up to now, only pure iron has been considered and studied for heat production through iron combustion (including liquid-state oxidation). Several combustion experiments have been performed at single-particle scale [6–9] as well as in multi-particle flames [10–13], allowing us to better understand the temperature evolution [8], size evolution [6] and gas production [14] associated with combustion. The main drawbacks currently encountered during iron combustion are (i) nanoparticle emission, (ii) micro-explosions and (iii) porosity formation and embrittlement of the combusted iron oxide products. $NO_{x}$ emission (iv) is also encountered due to the thermal dissociation of nitrogen and oxygen surrounding the burning particles, although Fe combustion is considered as a low-$\;NO_{x}$ emission process compared with fossil fuel combustion [14–17].

— (i) Although the flame temperature of iron is lower than its vaporization temperature, hematite nanoparticles are always produced when burned in stoichiometric and standard conditions in air (21% oxygen), together with micron-sized iron oxide particles [9,10,13]. The formation of nanoparticle clouds around burning particles was also observed by high-speed imaging during single-particle combustion, forming at temperatures close to the maximum flame temperature [8,12]. This nanoparticle cloud is no longer observed when decreasing the oxygen concentration of the surrounding gases to 14% [18], although nanoparticles can still be found in the combusted products for an oxygen concentration of 13.5% [17]. The quantification of nanoparticles produced during iron combustion was attempted by Wiinikka *et al*. [14] using a micro-cyclone and a low-pressure impactor, estimating 4 wt% of iron evaporation for an equivalence ratio of 0.5 and an oxygen concentration of 21%. Recently, Prasidha *et al*. [17] estimated a much lower range of nanoparticles emission of 0.01 to 0.13 wt% of the combusted products using a micro-cyclone and a HEPA filter, for an equivalence ratio ranging from 0.8 to 1.5 and an oxygen concentration between 13.5 and 21%.
— (ii) Micro-explosions of the liquid droplets are sometimes observed during the combustion of iron particles, occurring close to the maximum flame temperature. The frequency of these events increases with the oxygen concentration in the surrounding gases and is related to an increase of the flame temperature [8,19,20]. The exact origin of these micro-explosions is not fully understood. The main hypothesis is that they originate from the production or release of gas inside the liquid-burning particle [20–22]. A large volume of gas is produced inside the liquid iron/iron oxide droplets, forcing them to explode into smaller droplets to release the gaseous oxides. Micro-explosions are expected to deviate the particles from their initial flying path [8,19,20], as well as decrease the mean particle size of the products.
— (iii) The iron oxide particles formed as a product of the iron combustion are not dense but relatively porous, with pore sizes ranging from a few nanometres in diameter up to 80% of the particle diameter [13]. The presence of these pores is generally detrimental to the application of metal fuel as it decreases the energy density of the powders. Moreover, the presence of small pores can hinder the regeneration process of the combusted products. Non-percolating pores were indeed linked to water entrapment [23] and to the stabilization of iron oxide around these pores [24] during hydrogen-based direct reduction of iron oxide particles. Moreover, the formation of large spherical porosities induces easily fractured combusted products with thin oxide shells. The formation of these porosities was observed by high-speed imaging of single particles combustion, capturing a strong inflation of a few particles during their solidification [6]. While this inflation phenomenon is not fully understood yet, it is most likely due to the release of oxygen gas during the solidification process, due to the difference in oxygen solubility between the iron oxide liquid and the solid magnetite [13].
— (iv) $NO_{x}$ gases are classically formed from three different sources during fossil fuel combustion: thermal sources, from the thermal decomposition of oxygen and nitrogen gas; fuel sources, from the interaction between oxygen and the nitrogen content in the fuel; and prompt sources, where the hydrocarbons from the fuel combine with the molecular nitrogen in air in the flame front and facilitate $NO_{x}$ formation. In the case of pure Fe combustion, $NO_{x}$ gases are only produced from thermal sources. The $NO_{x}$emissions during the combustion of Fe have been measured in the range of close to 0 up to 37 mg/Nm³ of flue gas for an equivalence ratio ranging from 0.8 to 1.5 and an oxygen concentration between 13.5 and 21% [14]. $NO_{x}$ emissions have been estimated as more than 10–50 times lower in mass compared with the combustion of coal, for the same produced heat [14,17].

As mentioned, all existing studies on iron combustion (including liquid-state oxidation) focus on the use of pure iron powder, with the exception of the recent work from Peng *et al*. [25] studying the combustion of Fe powder containing 6.2 wt% Si. However, low-cost, impurities-containing iron powder is more interesting for this application to stay economically competitive. By decreasing the number of refinement steps required to obtain iron powders of high purity, we not only can decrease the cost of the iron powder but also the energy spent to produce the sustainable fuel. The enrichment step inside a blast oxygen furnace (BOF) following ores reduction in a blast furnace (BF), constituting the BF-BOF production route, is indeed associated with 190 to 230 kg of CO_2_ equivalent per ton of reduced metal and requires 150 to 350 kWh per ton of reduced metal [26,27]. This represents 11 to 13% of the total CO_2_ emissions and 4 to 10% of the energy consumed for the overall BF-BOF production route. In the other promising production route (DRI-EAF) based on a direct reduction of the ores (DRI) followed by an enrichment with an electric arc furnace (EAF), the enrichment step is associated with 250 to 650 kg of CO_2_ equivalent and requires 700 to 920 kWh per ton of reduced metal [4,26,27]. It represents 20 to 56% of the total CO_2_ emissions of the DRI-EAF production route and 15 to 27% of the total energy required. Moreover, the addition of selected impurities in iron could also solve some of the current drawbacks encountered during the combustion of pure iron powder.

The aim of this study is to propose a large screening of the expected tendencies from the most common impurities found in iron sources on their combustion through thermodynamic simulations. Its main goal is to facilitate the selection of the most promising or most problematic elements to be added to Fe fuel based on the best currently available thermodynamic databases (oxidation heat, energy density, oxygen solubility, vapour pressure). The impact of these impurities on the regeneration of the combusted products by direct reduction in fluidized bed reactors is also discussed to consider the influence of the impurities on the full metal fuel cycle.

## 2. Methods

## (a) Thermodynamic simulations

The combustion process begins with the ignition of the metallic particles. The ignition consists of a thermal runaway phenomenon, where the heat rate generated by the solid-state oxidation of the powder becomes superior to the heat rate exchange with the surrounding gases. Consequently, the temperature of the particle increases continuously, accelerating the oxidation kinetics and the heat production rate. When the temperature is sufficiently high, around 1080K for a pure iron particle with a diameter above 5 µm in air [28], oxidation kinetics are no longer dependent on the chemical reaction itself but dominated by the external diffusion of gaseous oxygen to the surface of the particle [29]. The oxidation process is then said to be in the diffusive regime [29]. In the diffusive regime, the flame temperature of a stoichiometric air–fuel mixture can be well approximated by an adiabatic system where all the heat from the oxidation process is used to increase the temperature of the particle as well as of the oxidizing gas [11,30,31]. A purely thermodynamic simulation of an adiabatic oxidation process can, therefore, be used to estimate the flame temperature during the combustion of iron particles when burning in a diffusive regime a stochiometric air–fuel mixture. It should be noted that a transition from a purely diffusive regime to a mixed regime was observed in the case of pure Fe combustion, although the nature and parameters of the new kinetic limiting mechanism for the oxidation process are still an ongoing research question [32–34]. In this work, a purely diffusive regime is considered to qualitatively compare the impact of impurities on the combustion of Fe-based powder.

The results presented in the manuscript were generated by FactSage thermochemical software version 8.3 [35] considering the adiabatic flame temperature of metal fuels under variable metal fuel to air ratios. Equilibrium calculations were performed with the commercial FactSage databases FSstel (metallic compounds and solutions), FToxid (oxide compounds and solutions) and FactPS (gaseous species, nitride and sulfide compounds). The commercial database FScopp was used in place of FSstel database for the equilibrium calculations with Cu impurity. These results were compared with the predictions obtained using Thermo-Calc with the TCOX11 database (ionic liquids and solid phases) and SSUB3 database (gaseous species), and are presented in the electronic supplementary material. FactSage and Thermo-calc software are both popular and powerful software that contain comprehensive thermodynamic properties of numerous compounds and solutions to perform multi-component and multi-phase equilibrium calculations. These thermodynamic databases are constructed using the CALPHAD method. This technique consists in parametrizing the Gibbs energy of each phase in a given system based on experimental data related to crystal structure, phase diagram, phase equilibria and thermodynamic data.

The adiabatic flame temperature calculations were performed considering the most stable assemblage of Fe with impurity at 298K and air (21 vol. % O_2_) at 298K as initial state. Equilibrium calculations assuming no heat loss outside of the considered system (including the particle and the remaining combusted gas) and 1 atm pressure were performed with incremental amounts of air to examine the evolution of the combustion reactions. At each air increment, the resulting combustion temperature, mass and composition of each phase are collected.

Two types of thermodynamic simulations have been conducted, denominated as (i) ‘global equilibrium’ and (ii) ‘step equilibrium’. In the global equilibrium system, a given amount of fresh air (21% oxygen and 79% nitrogen) is added at each simulation step, increasing the volume of the system to keep an atmospheric pressure. The oxygen added reacts to oxidize the different elements considered in the system, depending on their relative stability. The heat of oxidation is used to increase the temperature of both the particle and the combusted gases. It corresponds well to a diffusive flame with infinitely large mass diffusion coefficients in the gas phase and inside the particle. The combustion gases (mainly nitrogen) are accumulated in the system and are heated to the same temperature as the burning particle, i.e. as if the heat diffusion coefficients in the gas phase were also infinitely large. The flame temperature obtained in the global equilibrium system is a lower bound of the flame temperature reached in an ideal diffusive flame in stoichiometric and standard conditions, due to the large amount of gas needed to be heated to the maximum temperature of the particle. In reality, only a layer of the surrounding gas will be heated to the particle temperature, with a gradient of temperature in the gas phase decreasing from the particle surface. As the time scales of heat diffusion are not represented in this simulation, a second idealized thermodynamic simulation is considered instead. This second simulation represents a higher bound on the flame temperature obtained in an ideal diffusive flame. In the step equilibrium system, the gaseous products of the combustion are removed from the system at each simulation step and do not participate in subsequent equilibrium calculations. The volume of the step equilibrium system can be assumed to remain constant, with only a small amount of fresh air injected at each simulation step being heated to the particle temperature. The volume of the system only changes depending on the variation of the amount of oxygen absorbed by iron (oxide) and the amount of iron (oxide) evaporation, both being negligible compared with the total volume of the system. The main difference between the global equilibrium and step equilibrium simulations is, therefore, the different volumes of gas considered and heated to the particle temperature at each increment of added air. The total volume of air added to the system is yet similar in both cases.

Due to the different manipulations of the gas phase in both models, the extent of evaporation is examined differently. In the step equilibrium system, iron evaporation is quantified as the cumulated mass of evaporated iron in the combusted gases that are removed at each increment of added air. In the global equilibrium system, iron evaporation is quantified as the peak of Fe evaporation progressively accumulated with the combusted gas in the system. The amount of evaporation at each simulation step is dictated by the vapour pressure of Fe and the volume of the gases. The total volume of gases added to the system being identical in both cases, the differences in terms of Fe evaporation will be mainly dictated by the variation of Fe vapour pressure with the gas composition and temperature in both systems.

For the following step equilibrium system simulations, a fixed number of steps is chosen. This choice is justified by a trade-off between accuracy and computational costs both increasing with the number of steps. Starting with an initial value of 3.28 g of air per step for an initial mass of 100 g of metal fuel, the number of steps was doubled (the air mass increment is halved) again and again until the results (predicted temperature and evaporated iron) reached a relative accuracy above 98%, for an air mass increment of 0.82 g of air per step. This value is kept for all the following step equilibrium system simulations.

The kinetics of the reaction are not considered in these purely thermodynamic simulations, neither thermal nor mass diffusion. Therefore, the temperature is always homogeneous in the system at each simulation step, and the most stable phases are always expected to form, regardless of the diffusion required to form these components. Yet, the kinetics of the various phase transformations will depend on the mass diffusion of the various species in the solid, liquid and gas phases and on the particle morphology. In this work, a perfect mix of each element is considered, allowing the most stable phases to form at each step of the combustion. In reality, the spatial distribution of impurities inside the Fe particles and the morphology of the particle are expected to impact ignition (not covered in this work) as well as the oxidation, phase transformations and evaporation processes. For example, even when the oxide formed by an impurity is more stable than the iron oxide, iron will oxidize first if solid diffusion of oxygen through iron is required for the oxidation of the impurity (i.e. if the impurity is located in the core of the iron particle). This is a major point of study that will necessitate the derivation or experimental fit of the mass diffusion coefficients of each considered species in the various phases formed during the oxidation process, unfortunately not yet available.

### (b) Main impurities in Fe-rich materials

Two different sources of low-cost iron powder were considered in this study: primary sources (mining) and secondary sources (recycling).

The primary source comes from the extraction of iron ores. There are more than 1000 mineral species bearing iron, but only four different minerals are effectively used to produce iron: magnetite $Fe_{3}O_{4}$, hematite $Fe_{2}O_{3}$, goethite FeOOH and siderite $FeCO_{3}$ [36]. These minerals are mixed with other gangue minerals, such as quartz $SiO_{2}$, kaolinite $Al_{2}Si_{2}O_{5}{\left(OH\right)}_{4}$ and gibbsite $Al{\left(OH\right)}_{3}$ [36]. A total of 80–90% of worldwide production and reserves of iron-rich minerals are formed as ‘banded iron formations’, denominated with BIFs [36]. It consists of sedimentary deposits of layered Fe-rich minerals with other gangue minerals. It is found mostly in South Africa, Australia, Ukraine, Canada and Brazil. Other Fe-rich ore deposits can be found, such as magmatic ores in Sweden (from magmatic sources); oolitic iron in Australia; and carbonate iron in Austria, Bosnia and Algeria [36].

The secondary source comes from the recycling of Fe-based alloys, i.e. mostly from recycled steels. Two sources of recycled steel can be considered: on the one hand, chips and dust produced during low-cost steel machinery, processing and shaping; and on the other hand, recycled scraps collected from after-use vehicles, household and commercial appliances, as well as construction waste.

**Figure 1.**
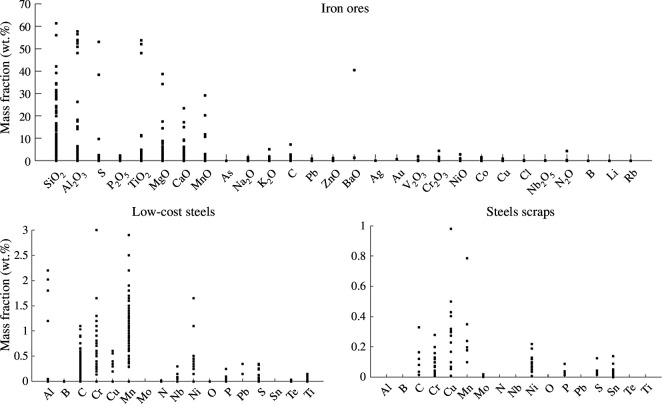
General composition of iron ores (for an iron content over 35 wt% Fe) [37–63], low-cost steels (less than 1.5 €/kg) [64] and steel scraps [65–67]. The main impurities in Fe-based sources are 𝑆𝑖𝑂_2_, 𝐴𝑙_2_𝑂_3_, 𝑆, 𝑇𝑖𝑂_2_, 𝑀𝑔𝑂, 𝐶𝑎𝑂, 𝑀𝑛𝑂 in iron ores; 𝐴𝑙, 𝐶, 𝐶𝑟, 𝑀𝑛, 𝑁𝑖 in low-cost steels and 𝐶, 𝐶𝑟, 𝐶𝑢, Mn, Mo, Ni, P and S in steel scraps.

The typical compositional ranges found in iron ores, low-cost steels and steel scraps are presented in figure 1. The main impurities considered in this work are Al,Cr,Cu,Mn,Mo,Ni,Si and Ti for the metallic elements, P,S and C for the light elements, and $Al_{2}O_{3},CaO,MgO,MnO,SiO_{2}$ and $TiO_{2}$ for the oxidized impurities. The general content in these different impurities differs depending on the source. To compare their relative influence on the combustion process, a fixed addition of 5 wt% was considered for each impurity.

### 3. Results and discussion

### (a) Pure iron combustion

Before investigating the role of impurities, the thermodynamic simulations of the combustion of pure iron powders were first compared with the reported experimental results.

**Figure 2.**
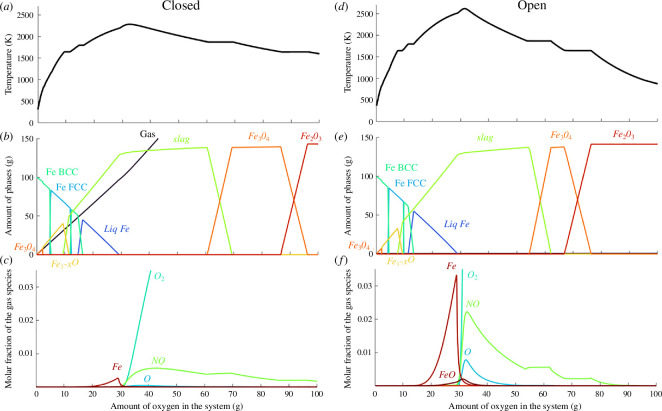
Thermodynamic simulation of the combustion of 100 g pure iron in air (21% O_2_ + 79% N_2_) considering (*a–c*) a global equilibrium system and (*d–e*) a step equilibrium system. Evolution of (*a,d*) the temperature of the system, (*b,e*) the phases stabilized, (*c,f*) the species in the gas phase. The phase evolution is similar in both systems, but a larger temperature and evaporation content is obtained in the step equilibrium system.

Figure 2 presents the thermodynamic simulation of the adiabatic iron combustion in air (at 21% $O_{2}$ + 79 at % $N_{2}$), in both global equilibrium and step equilibrium systems. As detailed in §2, none of these models perfectly represents what occurs in reality as the kinetics of heat and mass diffusion are not considered, but rather they give two boundaries on the amount of gas heated by the oxidation reaction, influencing both the particle temperature and the emission of gases. A range of about 350K is obtained for the prediction of the maximum temperature in both simulations (denominated with flame temperatures) for pure iron, equal to 2279K and 2633K in the step equilibrium and global equilibrium systems, respectively. This can be explained by the higher volume of gas added in the global equilibrium system, as the heat produced by the oxidation is used to increase the temperature of both the particle and the gas. Moreover, the step equilibrium system assumption leads to more iron evaporating. Indeed, the cumulated amount of iron evaporated in the step equilibrium system accounts for 0.73 wt%, while the maximum mass of iron in the gas only reaches 0.36 wt% in the global equilibrium system. The maximum amount of iron evaporation is predicted when the metallic liquid phase is fully transformed into the oxide liquid phase (slag), i.e. when the maximum temperature is reached in the metallic liquid phase. The larger amount of iron evaporation in the step equilibrium system can be justified by the larger vapour pressure reached in the fresh air added at each step, due to the larger temperature reached and lower Fe concentration in the gases.

The results from the simulations can be compared with the actual values measured experimentally during pure iron combustion. The simulated flame temperature range (2279–2633K) corresponds well to the flame temperature measured in air containing 21% oxygen by Ning *et al.* (2440K) [8]. A similarly good fit between the experimentally measured flame temperature and adiabatic simulation of a stoichiometric mixture was also reported for 20% oxygen concentration [30]. Yet other reported experimental flame temperatures (2000–2300K) [10,29] are lower than the thermodynamic predictions. McRae *et al.* [31] measured a flame temperature lower by 100K to the adiabatic thermodynamic prediction for an oxygen concentration of 30 and 40%, which was attributed to heat loss due to radiation. Another limitation could come from uncomplete ignition, or the use of non-stoichiometric mixtures (equivalence ratio of 1.2 in [10], and methane-air mixture with an equivalence ratio of 0.8 after full methane combustion in [29]). It could also come from the hypothesis of a diffusive flame. Thijs *et al.* [32], Fujinawa *et al.* [33] and Mich *et al*. [34] indeed argued that a mixed regime appeared at the end of the iron combustion, although the nature of the mechanisms limiting the oxidation process is unclear (oxygen adsorption; liquid-state oxygen diffusion in a core-shell structure; external boundary gas layer diffusion). The nature, concentration and spatial distribution of impurities inside the burning droplet will also influence the oxygen intake at the particle surface, as well as the internal oxygen diffusion. Therefore, the presence of impurities is expected to impact the transition from a diffusive to a mixed regime, slowing down the oxidation kinetics, limiting the flame temperature and associated oxygen concentration in the liquid oxide phase. The transition to a mixed regime has been hypothesized around an oxygen concentration of 50 at% [32–34] in the liquid oxide phase, although additional experimental characterization is required to confirm this value. In the thermodynamic simulations presented in this work, the oxidation rate is governed by the addition of air in the system, and the peak temperature is reached when the heat produced from the oxidation brought by the increment of oxygen added to the system becomes lower than the heat necessary to increase the temperature of all species in the system (mainly of the nitrogen in the combusted gas). It corresponds to an oxygen concentration in the liquid oxide phase of 52.8 at % O (in the global equilibrium simulation) and 52.4 at % O (in the step-equilibrium simulation) in the case of pure Fe.

Regarding the combusted products, both step equilibrium and global equilibrium simulations predict the full oxidization of iron to hematite ($Fe_{2}O_{3}$). Yet the actual combusted products collected at the end of iron combustion rather consist of a mix of magnetite and hematite phases [13]. The iron oxide liquid solidifies as magnetite and is further oxidized to hematite at the solid state, associated with slower oxidation kinetics compared with the liquid-state oxidation process. Therefore, the ratio between magnetite and hematite phases in the combustion products mainly depends on the cooling rate of the combusted powders.

The mass of $NO_{x}$ gas emissions is strongly overpredicted from the thermodynamic simulations (0.4–1.2 g/MJ) compared with the mass of $NO_{x}$ gas experimentally measured (0.01–0.03 g/MJ) [14]. These differences come from the relatively slow kinetics of $NO_{x}$ gas formation [15], which are considered as infinitely fast in these purely thermodynamic simulations. Consequently, the influence of impurities on the $NO_{x}$ emission gases is only represented qualitatively, in comparison with the predictions for a pure Fe combustion case. It corresponds to the hypothesis that the addition of impurities will not impact the kinetics of $NO_{x}$ formation. The presence of impurities, mostly inside the burning particle, is unlikely to impact the mechanisms of $NO_{x}$ formation in the surrounding gas phase in the case of a diffusive flame. Its main impact will be the modification of the flame temperature, hence of the gas temperature. The kinetics of $NO_{x}$ formation are expected to be accelerated with the gas temperature. Therefore, this work represents a lower bound on the expected influence of the addition of impurities on $NO_{x}$ emission.

Regarding nanoparticles emission, a range of 0.5–1.2 wt% of Fe evaporation is predicted here from the global equilibrium and step equilibrium systems. Prasidha *et al*. [17] measured around 0.1 wt% of Fe evaporation for a stoichiometric mixture in 21% oxygen. On the other hand, Wiinikka *et al*. [14] estimated the nanoparticles formation to 4 wt% of the combusted products, for an equivalence ratio of 0.5 in 21% oxygen. The strong variation in the experimental measurement could come from a difficulty of the collection of the nanoparticles in the combustion products. The thermodynamic simulations presented in this work cannot accurately quantify iron evaporation as the evaporation kinetics, mass diffusion of iron species in the gas phase and heat diffusion in the gas phase should be incorporated to quantify this phenomenon. Here, iron evaporation during the combustion of impure Fe powder will only be considered in comparison with what is predicted for pure Fe. The thermodynamic simulations highlight the influence of the impurities on the vapour pressure of Fe and on the temperature evolution of the particle in a diffusive flame, both influencing Fe evaporation. It should be noted that these comparisons are only valid as long as Fe evaporation kinetics are not strongly impacted by the impurities. This is a strong hypothesis as evaporation is mainly a surface phenomenon, hence could be strongly impacted by the spatial distribution of Fe in the particle. The hypothesis of similar Fe evaporation kinetics in impure powders is more reasonable if the impurities are considered to be concentrated in the centre of the burning particle. Yet strong variations from the predictions can be expected if the impurities are concentrated at the surface of the particle, in which case the evaporation kinetics of Fe are expected to be strongly lowered.

Finally, the predicted oxygen mass release during the solidification of the iron oxide liquid (from 0 to 0.27 g O/100 g Fe during magnetite solidification) corresponds to a maximum porosity size up to 98% of the particle diameter. The large porosities observed in the combusted products [13] could, therefore, well originate from oxygen release upon solidification.

### (b) Impurity-containing iron combustion

To compare the influence of the different impurities on the combustion process, several alloys containing 5 wt% of the selected impurities were considered. The detailed temperature and phase evolution predicted for each impurity can be found in the electronic supplementary material.

**Figure 3.**
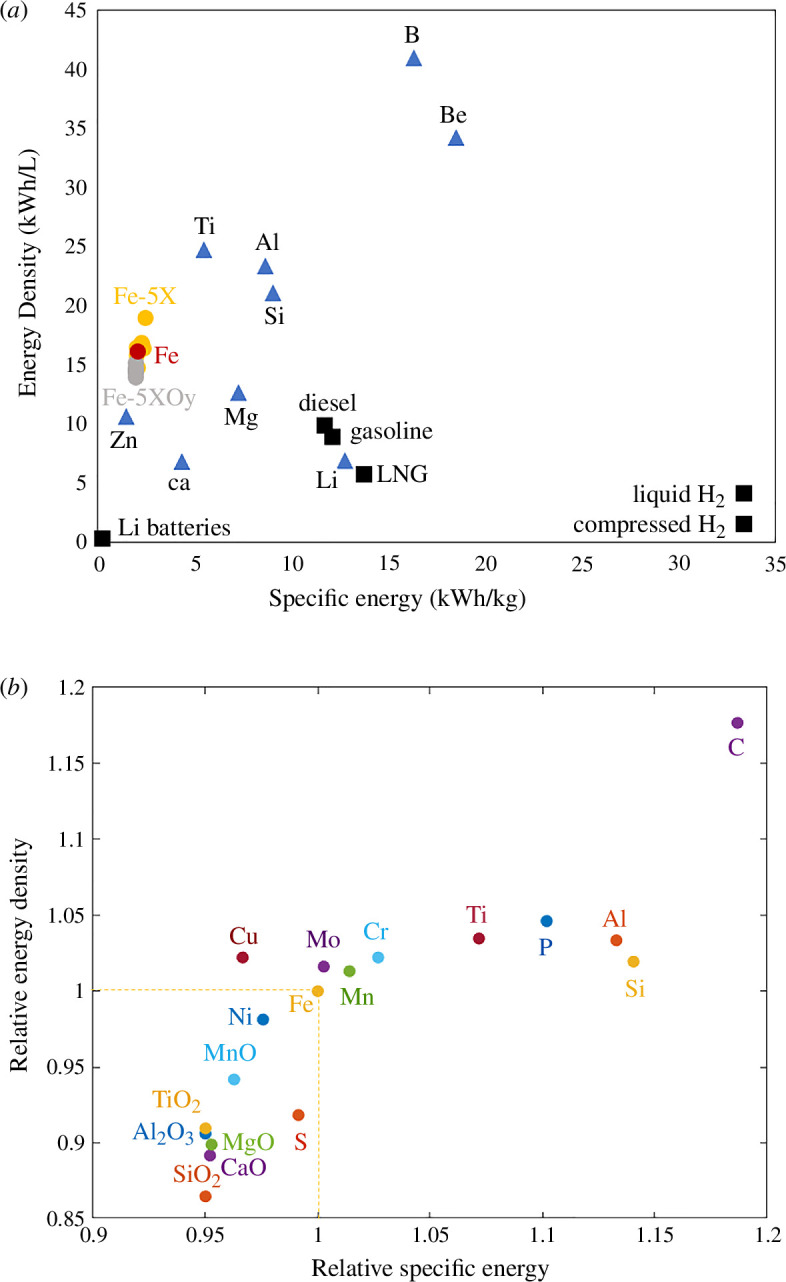
Influence of impurities on the energy density and specific energy of the Fe-based powder, when considering 5 wt% of each impurity, (*a*) compared with other energy-storing technologies and (*b*) relatively to pure iron. The oxidized impurities decrease the specific energy and the energy density of Fe-based powder while the metallic impurities and light elements increase them, except for Cu, Ni and S.

Figure 3 presents the influence of the presence of 5 wt% of impurities on the energy density and specific energy of Fe-based powder. Figure 3*a* compares the Fe-based powders with other considered fuels. Fe presents a lower energy density and specific energy compared with most other metals. Therefore, the addition of most metals to iron particles increases its energy density and specific energy, through the increase of its enthalpy of oxidation. On the other hand, the addition of already oxidized impurities decreases the energy density and specific energy of the Fe particles, as they are almost inert during the combustion process. Figure 3*b* presents the relative energy density and relative specific energy of the impurity-containing iron particles in relation to the ones of pure iron. The largest combination of energy density and specific energy is obtained with the addition of carbon, with an increase up to 20% as compared with pure Fe. As already highlighted in figure 3*b*, the addition of most metals increases the relative specific energy. A lower influence on the relative energy density is observed due to the combined change of the particle density together with the enthalpy of oxidation. A few notable exceptions are Cu, Ni and S. Cu and Ni present an enthalpy of oxidation lower than the one of Fe, decreasing the specific energy of Cu or Ni containing Fe powder. The energy density of Fe−5Cu is still slightly larger than the one of Fe due to its larger density (7.91 g/cm³) compared with pure Fe (7.86 g/cm³). In the case of S, the lower energy density and specific energy of S-containing Fe powder comes from the non-negligible enthalpy of formation of the initially formed FeS (4.34 kWh/kg), as well as from its lower density (7.24 g/cm³). The lowest energy density is obtained for $SiO_{2}$, with a decrease of nearly 15% as compared with pure iron, due to the strong decrease of its density (7.03 g/cm³). The lowest specific energies are obtained for $TiO_{2},SiO_{2}$ and $Al_{2}O_{3}$ with a decrease of 5% as compared with pure iron. These oxides are predicted to remain unchanged after the full oxidation of the Fe-based particles at room temperature, and are therefore considered as completely neutral in terms of total energy release during the combustion process. The larger specific energy obtained with the addition of MgO, MnO and CaO comes from their reaction with iron oxides, forming complex ternary oxides as the most stable phase at room temperature (see the electronic supplementary material). The current simulations consider the most stable phases formed for each oxygen concentration, i.e. a complete oxidation of the particle at the end of the oxidation process. As observed in the case of pure Fe [13], a complete oxidation at solid-state is unlikely, hence other complex ternary oxides could be found in the combusted products instead of the currently predicted pure iron oxides and Ti/Si/Al oxides.

**Figure 4.**
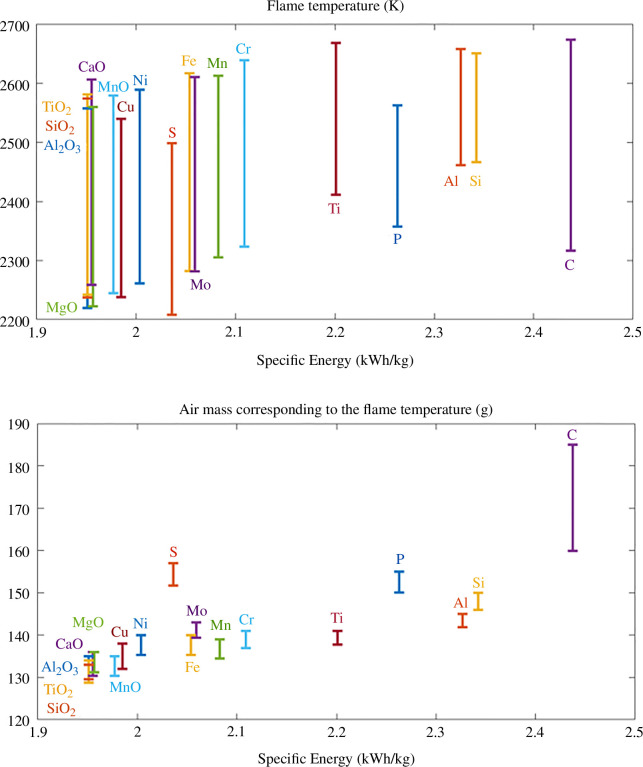
Range of flame temperature predicted in the adiabatic equilibrium and open system of an adiabatic combustion of Fe−5X [wt%], as a function of their energy density, as well as the associated air mass in the system. A general increase of the flame temperature is obtained with the specific energy, except for the light elements (S, P and C) due to the larger air mass added in the system at the maximum temperature.

Figure 4 presents the flame temperature predicted for the impurity-containing iron powders, as a function of their specific energy. A range of temperature is obtained based on the global equilibrium and step equilibrium systems, corresponding to the lower and higher bound for an ideal diffusive flame in stoichiometric mixture, respectively. As expected, a general increase of the flame temperature is observed with the specific energy. The exceptions are the light elements (S, P and C). Their lower flame temperatures are associated with a larger mass of air needed to fully oxidize the particles, as highlighted in figure 4*b*. It should be noted that these conclusions are only valid for a diffusive flame where the oxidation kinetics are limited by the external diffusion of oxygen to the surface of the burning particle. A decrease in flame temperature and increase in combustion time was recently reported by Peng *et al.* [25] during the combustion of Fe powders containing 6.2 wt% Si. The deceleration of the oxidation kinetics was explained by the limitation of oxygen intake, due to the formation of a Si-rich oxide layer at the surface of the burning particle reducing internal oxygen diffusion rate.

**Figure 5.**
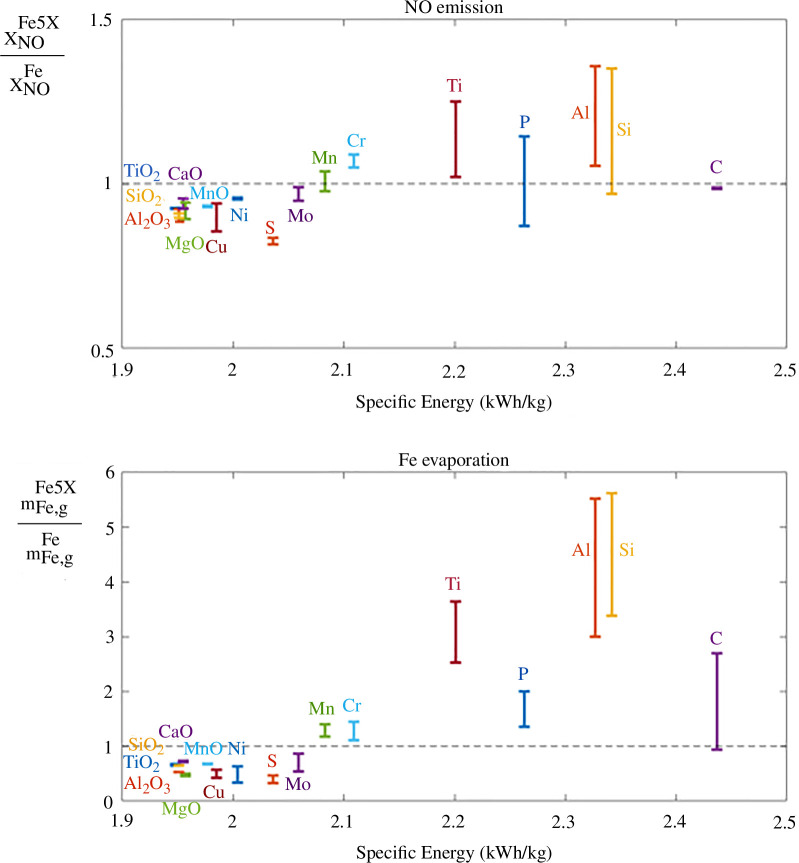
Predicted molar fraction of NO emission and mass of Fe evaporation in the combusted gas during the combustion of Fe−5X particles, normalized by the values obtained in the case of pure Fe, as a function of the energy density of Fe−5X. The same trend is observed for NO emission and Fe evaporation, following the flame temperature in figure 4.

In general, a larger flame temperature is preferred to obtain a larger efficiency of the heat transfer for energy recovery, but it is unfortunately associated with an increase in nanoparticles and $NO_{x}$ gases emissions, as presented in figure 5.

Figure 5 presents the influence of 5 wt% of different additions on the emission of NO gas as well as on evaporation of iron. As mentioned above, this figure relies on the strong hypothesis that the kinetics of NO formation and Fe evaporation are unimpacted by the addition of impurities in this work. However, these kinetics are expected to be mainly impacted by the modification of the flame temperature. The tendencies predicted by the thermodynamic simulations for NO emission and Fe evaporation would therefore be intensified by the modification of the kinetics of evaporation and NO formation with the gas temperature. Figure 5 presents the relative increase/decrease of NO emissions and Fe evaporation expected with the addition of impurities, compared with the case of pure Fe combustion.

A stronger influence of the impurities is observed for the evaporation of Fe, compared with NO emission. It can be explained by the additional influence of impurities on the activity of Fe in the liquid phase (as well as in the gas phase in the case of strong evaporation of the added element), modifying the vapour pressure of Fe. It can also be explained by the larger temperature reached at peak of Fe evaporation compared with the temperature reached at the peak of NO emission. NO emission comes from the thermal dissociation of oxygen and nitrogen in air, i.e. necessitate the presence of oxygen in the combusted gases. In the ideal case of a quasi-adiabatic diffusive flame, the oxygen introduced in the system quickly reacts in the oxidation process, hence only nitrogen is left in the combusted gas. In the thermodynamic simulations, NO only starts to form after the complete oxidation of the liquid oxide phase. In reality, a larger influence of increasing the flame temperature could be obtained as oxygen starts to dissociate and form $NO_{x}$ gases before reacting with the burning particles. Regarding iron evaporation, the maximum peak is always reached when the highest temperature is reached in the presence of metallic iron, i.e. at the end of the oxidation of the liquid metallic phase (as illustrated in figure 2).

**Figure 6.**
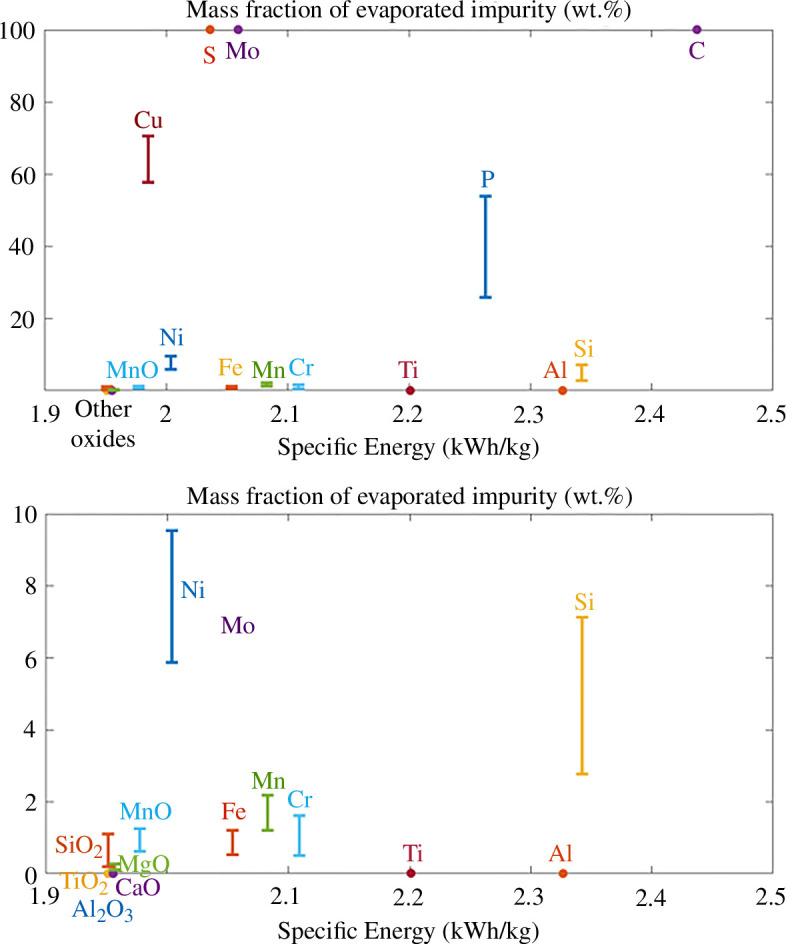
Comparison of the amount of X evaporation predicted during the combustion of Fe−5 wt % X, as a function of their respective energy density, at two different scales. Most impurities present a larger evaporation ratio as compared to iron, except for Al, Al2O3, Ti, TiO2, MgO and CaO. In particular, S, Mo and C are predicted to fully evaporate, and Cu and P to strongly evaporate.

Figure 6 presents the mass fraction of the added element in the gas phase. As for the prediction of Fe evaporation, the kinetics of the impurity evaporation is not considered in these purely thermodynamic predictions, predicting the vapour pressures in equilibrium in the combustion gases in a stoichiometric mixture. It represents the evaporation obtained when the evaporation kinetics are relatively fast compared with the oxidation kinetics, with fast mass diffusion of the evaporated species in the combusted gases. These values can be considered as a larger bound in the case of slower evaporation kinetics, due to the very fast oxidation kinetics or to the concentration of the impurities in the core of the burning particle (necessitating its mass diffusion to the surface of the liquid droplet to evaporate).

A complete evaporation of C, S and Mo is predicted, which agrees with the experimental observation of strong decrease in carbon content after combustion [13]. Up to 57–71% of the added Cu and 25–54% of the added P are also predicted to evaporate. Compared with the amount of iron nanoparticles produced during the combustion of pure iron, the addition of Ti, Al, Si, Mn, Ni and Cr would produce more nanoparticles, considering the evaporation of both Fe and the added metallic element. The larger propensity for Si to evaporate compared with Fe was confirmed in the recent work from Peng *et al.* [25], where a decrease in the relative content of Si compared with Fe was measured in the combusted products, compared with the Si-containing Fe particles.

The release of gaseous species during the combustion of iron powder has been related to micro-explosions events by several authors [11,20,68,69], and in particular related to the addition of CO in the system [68]. The release of large amounts of gaseous species during the combustion process, as expected from S, P, C and Mo, could therefore be problematic for this application. The combustion products will consist of a mix of gaseous species ($N_{2},O_{2},NO_{x},CO_{x},SO_{x}$), nanoparticles ($Fe_{2}O_{3}$, $P_{2}O_{5},MoO_{3},CuO,NiO,SiO_{2},\ldots )$ and micro-sized particles. Carbon oxides and sulfur oxides released during the combustion process should be captured if possible to limit their atmospheric pollution. If their concentration is too low to be efficiently captured, their presence in iron particles would decrease the sustainability of their use as metal fuel. However, it should be noted that their presence will only affect the first combustion cycle, as the regenerated combustion products would no longer contain carbon or sulfur. The rest of the evaporated elements will form nanoparticles by solidifying at lower temperatures. These nanoparticles could be captured from the gas phase using filters and be valorized or recycled, depending on the particle size, morphology and composition. Except for the case of Mn and Cr where a similar mass fraction of evaporation as iron is predicted, the collected nanoparticles should present a different concentration in impurities as compared with the initial material. A higher concentration in the impurity is expected for Cu, Ni, Mo, P and Si. On the other hand, a higher purity in Fe is expected in the nanoparticles formed with the presence of $SiO_{2},TiO_{2},Al_{2}O_{3},CaO,Ti,Al,S$ and C as initial impurities.

**Figure 7.**
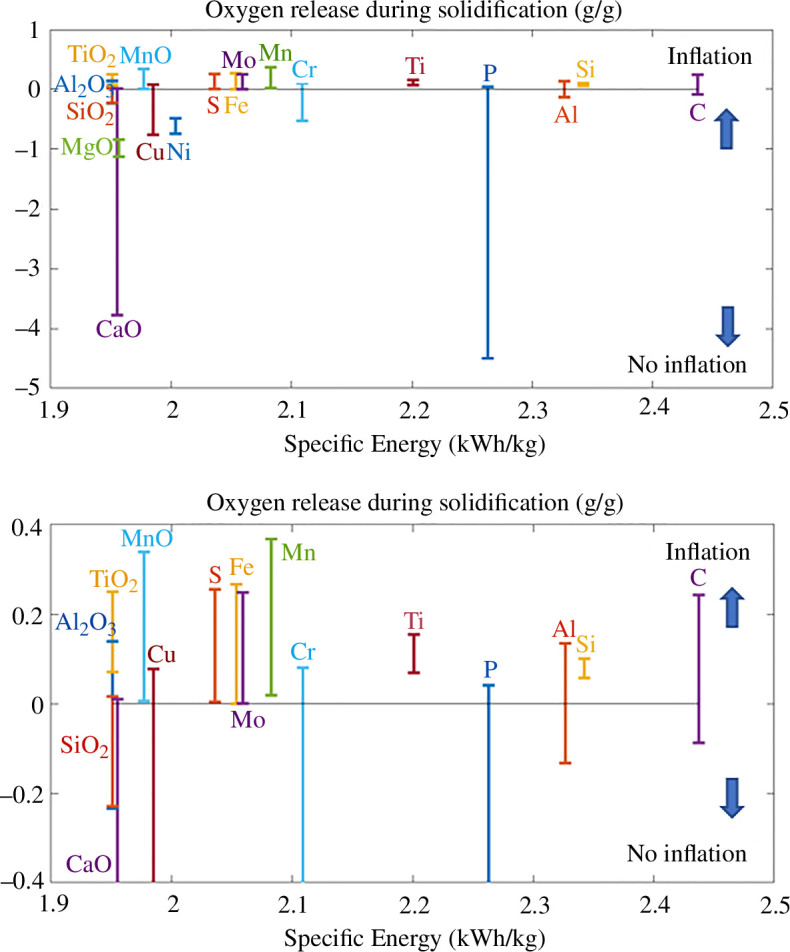
Comparison of the mass of oxygen release during the solidification of the slag, at the end of the combustion process of Fe−5 wt% X, as a function of their respective energy density. If oxygen is released (positive values of mass release), pores are expected to form in the solidified oxide powder. On the other hand, no inflation of the particle is expected if no oxygen gas is released during the solidification process. A zoom of the transition between the negative and positive mass release is given. No inflation is predicted for the addition of Ni, MgO and 𝑆𝑖𝑂_2_, while the addition of Mn or MnO is predicted to worsen the inflation of the combusted products.

Figure 7 presents the evolution of the oxygen mass release predicted during the solidification of the slag, at the end of the combustion process of Fe−5X powder. When the oxygen solubility of the slag is larger than the one of the first solid to form, as in pure iron, oxygen gas is released upon solidification. Such gas release has been associated with strong particle inflation during the solidification of pure iron powders [6]. As a consequence, large porosities are formed in the combusted products of iron combustion, decreasing their energy density and embrittling the combustion products consisting of thin oxide shells. A larger oxygen mass release is associated with a stronger inflation of the solidifying particle. Negative oxygen release values correspond to the suppression of the inflation phenomenon, as the oxygen solubility of the solid products is larger than the oxygen solubility of the slag. The complete suppression of the inflation phenomenon is predicted with the addition of $SiO_{2}$, CaO, MgO and Ni. Most impurities are predicted to also decrease the oxygen mass release as compared with pure Fe powder, except for the addition of $TiO_{2}$, Ti, MnO, Mn, Si, Mo, S and C. Peng *et al.* [25] reported an increase in porosity content in the combusted products of Fe powder containing 6.2 wt% Si, which could be related to an increase in oxygen gas release upon solidification, although this phenomenon should be studied in more detail to confirm this hypothesis.

The collected micro-scale oxidized particles could be directly reduced and reused for the application of metal fuel. Many different techniques of iron oxide reduction exist. In the case of metal fuels, solid-state reduction is preferred as it saves an additional and energetically costly atomization step from the reduced iron liquid to form the micro-scale powder needed for the combustion process. In particular, hydrogen-based direct reduction is currently the favoured reduction route for the concept of metal fuel as it is fully sustainable with no ${CO}_{2}$ emissions if hydrogen is sustainably produced. Moreover, the large-scale direct reduction of iron oxide is technologically more mature than other considered reduction routes, such as direct iron electrolysis. Hydrogen-based direct reduction of fine iron oxide particles requires a constant movement of the particles to avoid their sintering, for example in fluidized bed reactors. The main issue faced during the reduction of iron oxide fines in fluidized bed reactors is the defluidization of the powders obtained due to the agglomeration of the reducing particles, at the contact of the freshly reduced iron layer at the surface of the particles. It was observed that the addition of a fine oxidized powder such as $TiO_{2},Al_{2}O_{3}$, MgO or CaO together with the iron oxide particles was greatly hindering the agglomeration of the particles and postponing the defluidization [70]. The addition of MgO was reported to be more efficient than CaO to postpone defluidization [71]. A similar positive effect could be expected from the presence of these oxides inside the combusted products, if these oxides are present at the surface of the particles at the end of the combustion process. The presence of all elements less noble than iron (staying oxidized during the reduction of iron oxide) is expected to postpone defluidization. Among the impurities considered in this study, the addition of Al, CaO, Cr, Mn, MgO, Si or Ti can be expected to hinder defluidization. On the contrary, the remaining Cu or Ni in the combusted powder is more noble than iron and presents lower melting temperature, hence favoured sintering. Their presence could have a detrimental effect on the agglomeration of the particles and accelerate the defluidization of the powder during their reduction process, although it should be investigated experimentally. Finally, the initial presence of C, S or Mo in the particles will not influence their reduction process as these impurities are predicted to completely evaporate during the combustion step.

Regarding the reduction kinetics, a positive influence of Cu was observed in ternary Fe-Al-Cu oxides because of the increased nobility [72]. A similar positive influence is expected with the addition of Ni, for its increased nobility compared with pure iron. A positive influence on the reduction kinetics was also reported with the addition of CaO and MgO, notably due to the increased porosity during the reduction process, allowing a better penetration of the reductant gas to the core of the particles [73]. On the other hand, the addition of Ti and Mn are expected to slow down the reduction kinetics due to the stabilization of magnetite [73,74]. Reduction of 1.7 wt% Mn-containing wustite was reported to follow similar kinetics as pure wustite at 1223K [75]. However, Mn becomes concentrated in the unreduced wustite phase at the end of the reduction process and finally precipitates as (Mn,Fe)O [75]. In the case of $SiO_{2}$, a first positive influence was reported for a concentration lower than 0.5%, then a deceleration of the reduction kinetics was observed at the end of the reduction process [73]. In the case of $Al_{2}O_{3}$, its effect on magnetite reduction depends on the alumina content and temperature [76]. At 1023K under CO, reduction kinetics are accelerated by the addition of 3 wt% alumina, but decreased with a further increase of the alumina content to 6–12 wt% [76]. At 1123K, the fastest reduction kinetics are obtained for 6 wt% alumina [76]. The positive effect was observed due to the lower density of the iron shell, compared with the reduction of pure magnetite, and to the formation of cracks during the reduction of the mixed magnetite and $FeAl_{2}O_{4}$ phase mixture [76]. The addition of 5–20 at % Al stabilizes magnetite and postpones its reduction when reduced in a mix of Ar/$H_{2}$ gas during TPR at 10 K/min to 1223K [72]. Addition of 3 at % Cr, Al and Ti slows down the reduction at 600K under 33.3 kPa of hydrogen [77]. The negative effect of these additives decreases with cycling and the addition of Cr was even positive on the reduction kinetics after three redox cycles, the origin of which was not linked to a surface area change and could not be explained in that study [77].

The influence of the considered impurities on the combustion and regeneration process through direct reduction in fluidized bed reactors is summarized in table 1, in comparison with the combustion and regeneration of pure iron powder. Light elements such as C, P and S are interesting in terms of decreasing $NO_{x}$ and Fe nanoparticles emissions for a larger specific energy, but they are also associated with strong gas emissions, hence expected micro-explosions, and environmentally harmful gases in the case of C and S. Cu addition is also interesting in terms of limiting Fe evaporation and decreasing $NO_{x}$ gas, and could be considered as an interesting path for scrap purification as Cu is a particularly problematic tramp element in recycled steels. The addition of Al and Si will efficiently increase the energy density of the powder, but strongly increase Fe evaporation, as well as $NO_{x}$ gas. Among the oxidized impurities, the addition of CaO and MgO are particularly interesting in terms of limiting Fe evaporation and $NO_{x}$ gas, while hindering oxygen release during the powder solidification and improving the fluidization as well as reduction kinetics of the combusted powder for its regeneration.

**Table 1.** Summary of the expected impact of the considered impurity X in Fe−5 wt% X on the specific energy (kWh/kg), energy density (kWh/l), flame temperature $T_{f}$ (K), NO gas (atm), iron evaporation Fe (g), impurity evaporation X (g), oxygen release (at the origin of the porosities) O (g), on the reduction kinetics and on the fluidization of the powders in fluidized bed reactors. The influence is qualitatively evaluated with ‘+‘ and ‘−‘ when positively or negatively impacted, or ‘~‘ when similar results as for pure iron are expected. ‘+/−‘ is used for the reduction kinetics and fluidization process when it depends on the experimental procedure, and ‘/‘ signifies that no pertinent data were found. ‘NA’ stands for ‘not applicable’ when the impurity is predicted to fully evaporate previous to the regeneration process.

impurity X	specific energy	energy density	$T_{f}$	NO	Fe(g)	X(g)	O(g)	reduction	fluidization
C	+ + +	+ + +	+	−	+ +	+ + + + +	−	NA	NA
P	+ +	+	~	~	+	+ + +	− − −	/	/
S	−	− −	− −	− −	− −	+ + + + +	−	NA	NA
Mo	~	+	~	−	−	+ + + + +	−	NA	NA
Cu	−	+	−	− −	− −	+ + +	− − −	+ +	−
Ni	−	−	−	−	− −	+ +	− − − −	+ +	−
Cr	+	+	+	+	+	+	− − −	+/−	/
Al	+ +	+	+ +	+ + +	+ + + +	− − −	− −	+/−	+
Si	+ +	+	+ +	+ + +	+ + + +	+ +	− − −	+/−	+
Ti	+	+	+ +	+ +	+ + +	− − −	− −	−	+
Mn	+	+	+	~	+	+	+ +	−	+
MgO	−	− −	−	− −	− −	− −	− − − −	+	+ +
MnO	−	−	−	−	−	~	+	−	+
$Al_{2}O_{3}$	−	− −	−	− −	−	− − −	− −	+/−	+
${SiO}_{2}$	−	− − −	−	− −	−	−	− − − −	+/−	+
${TiO}_{2}$	−	− −	−	−	−	− − −	−	− −	+
CaO	−	− −	−	−	−	− − −	− − −	+	+

## 4. Summary and outlook

A large screening of the positive or negative influence of the main impurities found in iron-sources on their use as iron fuel was conducted in this work using purely adiabatic thermodynamic simulations. In particular, this study focuses on the influence of 5 wt% of various impurities (C, P, S, Mo, Cu, Ni, Cr, Al, Si, Ti, Mn, MgO, MnO, $Al_{2}O_{3}$, ${SiO}_{2}$, and CaO) on the flame temperature obtained in an ideal diffusive flame in stoichiometric mixture, and on the associated Fe evaporation, impurity evaporation, NO emissions and oxygen release upon solidification, considered as at the origin of the large porosities observed in the pure combusted iron particles.

For each property, a range of values was obtained considering two different thermodynamic simulations: one system in which the combusted gases accumulate during the oxidation process, of increasing volume and constant atmospheric pressure (lower bound of flame temperature); and a step equilibrium system in which the combusted gases from the previous simulation step are evacuated from the system to keep a constant volume and atmospheric pressure, i.e. as if a constant air flux was flushed around the burning particle (higher bound of flame temperature).

In the case of pure Fe combustion, the flame temperature of a diffusive flame in stoichiometric air–fuel mixtures and standard conditions is well represented by an adiabatic thermodynamic simulation and plausible values are obtained for the oxygen release upon solidification. However, NO emissions and evaporation processes are overestimated due to the slow associated kinetics. This work is not intended as an accurate predictive tool but rather a guide towards the expected impact of impurities on its thermodynamic properties and associated combustion behaviour. The impact of impurities on NO emissions and Fe evaporation are therefore expressed in relation to the values obtained for pure Fe, showing a positive or negative expected influence on these processes. The thermodynamic simulation of the adiabatic combustion of Fe−5 wt% X (where X is the considered impurity) in the global equilibrium system was conducted in both FactSage and Thermo-calc software to compare and validate the database used (see the electronic supplementary material).

The additions of C, Al or Si are very interesting in terms of specific energy and energy density, but are associated with a large volume of $CO_{2}$ emissions and micro-explosions in the case of carbon for the first combustion cycle, and with an increase of iron evaporation and $NO_{x}$ emissions in the case of Al and Si. According to the thermodynamic simulations, the addition of S is the most efficient to decrease $NO_{x}$ emissions and iron evaporation due to the relatively larger amount of air required to fully oxidize S, but is associated with the emission of toxic $SO_{x}$ gases. As for carbon, sulfur is expected to be completely separated from iron after the first combustion cycle. The use of Fe-based powder as metal fuel can also be considered as an interesting purification process in the case of P, Mo, Cu and Ni, as these impurities are expected to strongly evaporate during the combustion process.

The additions of MgO or CaO are the most interesting additions in terms of suppressing the porosities in the combusted products, limiting nanoparticles emission, and improving the regeneration process in fluidized bed reactors. However, they are associated with a decrease of the energy density and the specific energy of the fuel.

Future works will focus on the experimental investigation of combustion and regeneration of impure Fe particles containing these selected promising impurities. The obtained data will hopefully provide the currently missing kinetic parameters (mass and heat diffusion coefficients, associated oxidation and evaporation kinetics) and morphological data (space distribution of iron and the impurity in the solid and liquid phases) required to derive a predictive model of the combustion of impure Fe powders.

## Data Availability

The data presented in this manuscript come from the database of both Thermocalc and FactSage software. The graph of the detailed data is given in the supplementary materials [78].
